# Early Prediction of Response Focused on Tumor Markers in Atezolizumab plus Bevacizumab Therapy for Hepatocellular Carcinoma

**DOI:** 10.3390/cancers15112927

**Published:** 2023-05-26

**Authors:** Norikazu Tanabe, Issei Saeki, Yuki Aibe, Takashi Matsuda, Tadasuke Hanazono, Maiko Nishi, Isao Hidaka, Shinya Kuwashiro, Shogo Shiratsuki, Keiji Matsuura, Maho Egusa, Natsuko Nishiyama, Tsuyoshi Fujioka, Daiki Kawamoto, Ryo Sasaki, Tatsuro Nishimura, Takashi Oono, Takuro Hisanaga, Toshihiko Matsumoto, Tsuyoshi Ishikawa, Takahiro Yamasaki, Taro Takami

**Affiliations:** 1 Division of Laboratory, Yamaguchi University Hospital, Ube 755-8505, Yamaguchi, Japan; norikazu@yamaguchi-u.ac.jp (N.T.); t.yama@yamaguchi-u.ac.jp (T.Y.); 2 Department of Gastroenterology and Hepatology, Yamaguchi University Graduate School of Medicine, Ube 755-8505, Yamaguchi, Japan; egusa@yamaguchi-u.ac.jp (M.E.); nabo312@yamaguchi-u.ac.jp (N.N.); tsufuji@yamaguchi-u.ac.jp (T.F.); dkawa@yamaguchi-u.ac.jp (D.K.); ryo0530@yamaguchi-u.ac.jp (R.S.); ntatsuro@yamaguchi-u.ac.jp (T.N.); toono@yamaguchi-u.ac.jp (T.O.); t-hisa01@yamaguchi-u.ac.jp (T.H.); tm0831@yamaguchi-u.ac.jp (T.M.); tsu0920@yamaguchi-u.ac.jp (T.I.); t-takami@yamaguchi-u.ac.jp (T.T.); 3 Yamaguchi Clinical Research Group—Hepatology (YCR-H), Ube 755-8505, Yamaguchi, Japanm-nishi@yamaguchih.johas.go.jp (M.N.); ihidaka@yamaguchi.saiseikai.or.jp (I.H.); shinya-k@wish.ocn.ne.jp (S.K.);; 4 Department of Gastroenterology, Kokura Memorial Hospital, Kitakyushu 802-8555, Fukuoka, Japan; 5 Department of Gastroenterology, Shimonoseki Medical Center, Shimonoseki 750-0061, Yamaguchi, Japan; 6 Department of Gastroenterology, Saiseikai Shimonoseki General Hospital, Shimonoseki 759-6603, Yamaguchi, Japan; 7 Department of Gastroenterology, Yamaguchi Rosai Hospital, Sanyo-Onoda 756-0095, Yamaguchi, Japan; 8 Department of Gastroenterology, Saiseikai Yamaguchi General Hospital, Yamaguchi 753-0078, Yamaguchi, Japan; 9 Department of Gastroenterology, Yamaguchi Prefectural Grand Medical Center, Hofu 747-8511, Yamaguchi, Japan; 10 Department of Gastroenterology, Tokuyama Central Hospital, Syunan 745-8522, Yamaguchi, Japan; 11 Department of Gastroenterology, Shuto General Hospital, Yanai 742-0032, Yamaguchi, Japan; 12 Department of Oncology and Laboratory Medicine, Yamaguchi University Graduate School of Medicine, Ube 755-8505, Yamaguchi, Japan

**Keywords:** hepatocellular carcinoma, atezolizumab, bevacizumab, alpha-fetoprotein, des-gamma-carboxy prothrombin, immune checkpoint inhibitor

## Abstract

**Simple Summary:**

The combination of atezolizumab and bevacizumab was introduced as a first-line therapy for patients with unresectable hepatocellular carcinoma in 2020. Although some patients have shown a treatment response, there have also been those with disease progression. Such cases should be appropriately transitioned to second-line or later treatment. Thus, this study investigated early predictors of response and disease progression categorized into two groups based on a baseline alpha-fetoprotein (AFP) of 20 ng/mL. As a result, we found that changes in AFP and baseline des-gamma-carboxy prothrombin levels were useful predictors of treatment response. Tumor markers are useful in predicting treatment response and prognosis.

**Abstract:**

Despite the promising efficacy of atezolizumab plus bevacizumab (atezo/bev), some patients with unresectable hepatocellular carcinoma (HCC) experience disease progression. This retrospective study, which included 154 patients, aimed to evaluate predictors of treatment efficacy of atezo/bev for unresectable HCC. Factors associated with treatment response were examined, focusing on tumor markers. In the high-alpha-fetoprotein (AFP) group (baseline AFP ≥ 20 ng/mL), a decrease in AFP level > 30% was an independent predictor of objective response (odds ratio, 5.517; *p* = 0.0032). In the low-AFP group (baseline AFP < 20 ng/mL), baseline des-gamma-carboxy prothrombin (DCP) level < 40 mAU/mL was an independent predictor of objective response (odds ratio, 3.978; *p* = 0.0206). The independent predictors of early progressive disease were an increase in AFP level ≥ 30% at 3 weeks (odds ratio, 4.077; *p* = 0.0264) and the presence of extrahepatic spread (odds ratio, 3.682; *p* = 0.0337) in the high-AFP group and up-to-seven criteria, OUT (odds ratio, 15.756; *p* = 0.0257) in the low-AFP group. In atezo/bev therapy, focusing on early AFP changes, baseline DCP, and tumor burden of up-to-seven criteria are useful in predicting response to treatment.

## 1. Introduction

The combination therapy of atezolizumab (programmed death 1 inhibitor) plus bevacizumab (vascular endothelial growth factor antibody drug) for treating unresectable hepatocellular carcinoma (HCC) was introduced in 2020. For a long time, the tyrosine kinase inhibitors sorafenib and lenvatinib have been previously recommended as first-line therapies for unresectable HCC; however, as immuno-oncology treatment, atezolizumab plus bevacizumab (atezo/bev) (IMbrave150 trial) and tremelimumab plus durvalumab (HIMALAYA trial) demonstrate a significant improvement in overall survival versus sorafenib [1,2]. Currently, atezo/bev therapy is used as first-line treatment for unresectable HCC in many cases [3,4,5,6].

Despite the high response rate of 27.3% (Response Evaluation Criteria in Solid Tumors [RECIST] 1.1) in the IMbrave150 trial, 19.6% of patients had progressive disease (PD) at the initial imaging evaluation. A similarly high response rate has also been reported in real-world clinical practice; however, early PD has also been observed [7,8,9,10]. Currently, the available second-line and beyond agents include sorafenib, lenvatinib, regorafenib, ramucirumab, and cabozantinib. Although the appropriate subsequent treatment after atezo/bev therapy has not been established [3,5], prolonged overall survival (OS) has been reported in cases where tyrosine kinase inhibitors were administered after using immune checkpoint inhibitors as primary therapy [11]. Therefore, appropriate sequential therapy is considered important. This study aimed to identify early indicators of response to atezo/bev therapy.

## 2. Materials and Methods

### 2.1. Study Design and Patients

Overall, 205 patients who received atezo/bev therapy for unresectable HCC at our hospital and affiliated institutions between September 2020 and November 2022 were evaluated in this multicenter retrospective study. The exclusion criteria were no imaging evaluation and missing data on tumor marker levels. HCC was diagnosed based on imaging, pathological findings, alpha-fetoprotein (AFP) levels, and des-gamma-carboxy prothrombin (DCP) levels according to the Japanese guidelines [2]. The staging was based on the Barcelona Clinic Liver Cancer (BCLC) staging system [4].

### 2.2. Treatment Protocol and Evaluation of Treatment Response and Tumor Markers

Patients received 1200 mg atezo and 15 mg/kg bev every 3 weeks. Atezo/bev therapy was administered until radiological PD or intolerable adverse events occurred. Initial radiological evaluation using computed tomography was performed 6 weeks after initiating atezo/bev therapy (the day of the third atezo/bev). Treatment response was evaluated using RECIST 1.1 [12]. Objective response rate (ORR) was defined as the percentage of patients with complete response (CR) and partial response (PR), and disease control rate (DCR) was defined as the percentage of patients with CR, PR, and stable disease (SD). AFP levels were measured 3 weeks after initiating treatment (the day of the second atezo/bev).

### 2.3. Statistical Analysis

Data are presented as median and quartiles. Comparisons of treatment effects were performed using the chi-square test. Progression-free survival (PFS) was defined as the time from the date of atezo/bev administration to the date of radiological tumor progression or any cause of death. OS was defined as the time from the date of atezo/bev administration to the date of any cause of death. Patients who were lost to follow-up were censored at the last visit, and those who were alive on 31 December 2022 were censored. PFS and OS were analyzed using the Kaplan–Meier method and compared between groups using the log-rank test. Spearman’s test was used for the correlation analysis between two continuous variables. Furthermore, the best cutoff value for the change in AFP level was determined using the receiver operating characteristic (ROC) curve. For the prediction of objective response (OR), the Youden index was used to set the cutoff value, and for the prediction of early PD, a cutoff value was set based on 80% specificity. To define predictive factors of OR and early PD, we evaluated the following factors: age, sex, Eastern Cooperative Oncology Group performance status, etiology, treatment line, modified albumin-bilirubin score (mALBI) grade [13], BCLC stage, macrovascular invasion (MVI), extrahepatic spread (EHS), tumor burden of up-to-seven criteria [14], and tumor markers. Univariate and multivariate analyses of factors contributing to OR and early PD were performed using logistic regression analysis, and the results were presented as odds ratios with a 95% confidence interval (CI). Moreover, univariate and multivariate analyses of factors contributing to PFS and OS were performed using the Cox proportional hazards model, and hazard ratios and their 95% CIs were calculated. The selection of factors for multivariate analysis was based on liver function and tumor-related factors including tumor markers, regardless of *p*-values based on univariate analysis. Statistical significance was set as *p* < 0.05. All statistical analyses were performed using the JMP software package v16.0 (SAS Institute, Cary, NC, USA).

## 3. Results

### 3.1. Patient Characteristics

**Figure 1:**
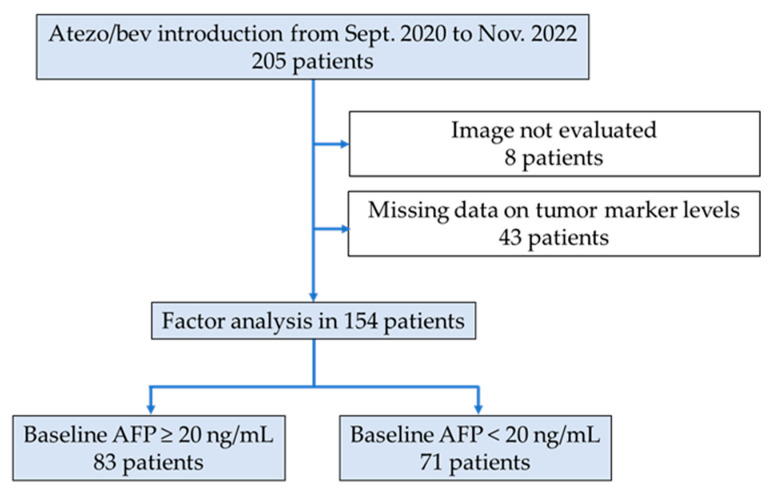
Study chart. Overall, 205 patients were treated with atezolizumab plus bevacizumab at various centers. Fifty-one patients were excluded because of insufficient data. They were categorized into the high-AFP (baseline AFP ≥ 20 ng/mL; *n* = 83) and low-AFP (baseline AFP < 20 ng/mL; *n* = 71) groups. atezo/Bev, atezolizumab plus bevacizumab; AFP, alpha-fetoprotein.

Among the 205 patients, 51 were excluded because they did not undergo imaging evaluation (*n* = 8) or data on tumor marker levels were missing (*n* = 43) (Figure 1). Finally, 154 patients were included in this study. Baseline patient characteristics are summarized in Table 1. The median patient age was 75 years; 124 (80.5%) patients were male; and 17 (11.0%), 52 (33.8%), and 85 (55.2%) patients had hepatitis B virus infection, hepatitis C virus infection, and non-viral causes of HCC, respectively.

The mALBI grades were 1, 2a, 2b, and 3 in 46 (29.9%), 38 (24.7%), 68 (44.2%), and 2 (1.3%) patients, respectively. BCLC stages were A, B, and C in 11 (7.1%), 79 (51.3%), and 64 (41.6%) patients, respectively. Overall, 45 (29.2%) and 29 (18.8%) patients had MVI and EHS, respectively. The median number and size of the tumors were 4 and 30 mm, respectively. The median AFP and DCP levels were 31.1 ng/mL and 382.4 mAU/mL, respectively. Atezo/bev therapy was administered as first- and later-line treatments in 99 (64.3%) and 55 (35.7%) patients, respectively. The median duration of follow-up was 10.2 months (95% CI, 5.2–15.9).

### 3.2. Relationship between Treatment Response and Change in AFP Levels

The best treatment responses according to RECIST 1.1 for CR, PR, SD, and PD were 9 (5.8%), 46 (29.9%), 65 (42.2%), and 34 (22.1%) patients, respectively. ORR and DCR were 35.7% and 77.9%, respectively (Table 2). The median OS and PFS were 18.5 months (95% CI, 15.2–NE) and 7.8 months (95% CI, 6.5–10.5), respectively (Figure S1).

We examined predictors of treatment response separately in the high-AFP (baseline AFP ≥ 20 ng/mL; *n* = 83) and the low-AFP (baseline AFP < 20 ng/mL; *n* = 71) groups (Figure 1). We used 20 ng/mL as the cutoff value, which was suggested by the American Association for the Study of Liver Diseases [15]. The respective group characteristics are presented in Table 1. In this study, we focused on changes in AFP levels at 3 weeks, which is earlier than the 6 weeks imaging response assessment. The changes in AFP levels at 3 weeks and those at 6 weeks were positively correlated, regardless of the groups (high-AFP group, r = 0.880, low-AFP group, r = 0.805) (Figure S2).

**Figure 2:**
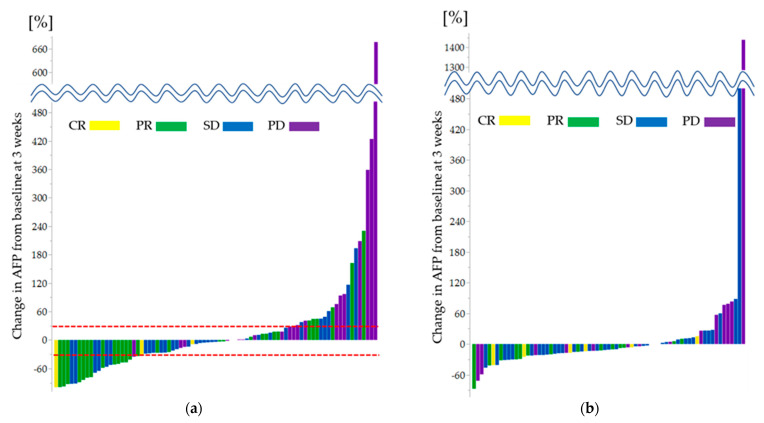
Waterfall plot of change in AFP levels from baseline at 3 weeks and best treatment response per RECIST 1.1 in (**a**) the high-AFP group and (**b**) the low-AFP group. AFP, alpha-fetoprotein; CR, complete response; PR, partial response; SD, stable disease; PD, progressive disease; RECIST, Response Evaluation Criteria in Solid Tumors.

Figure 2 shows the waterfall plot of the relationship between the best imaging treatment response and changes in AFP levels from baseline to 3 weeks after treatment initiation for (a) the high-AFP and (b) low-AFP groups, respectively.

### 3.3. Predictor of OR Outcome in the High-AFP Group

In the high-AFP group, the cutoff value for OR was set at a decrease in AFP level > 30% from baseline to 3 weeks after treatment initiation (sensitivity, 51.6%; specificity, 86.0%; and area under the curve (AUC), 0.659 by the ROC curve analysis with Youden index). As shown in Figure 2a, many of the patients with a decrease in AFP level > 30% achieved OR. The results of the univariate and multivariate analyses for OR are presented in Table 3. Univariate analysis showed that a decrease in AFP level > 30% was associated with OR (odds ratio, 5.782; 95% CI, 2.022–16.535; *p* = 0.0011). Consistently, multivariate analysis showed that a decrease in AFP level > 30% was an independent influencing factor of OR (odds ratio, 5.517; 95% CI, 1.773–17.165; *p* = 0.0032).

**Figure 3:**
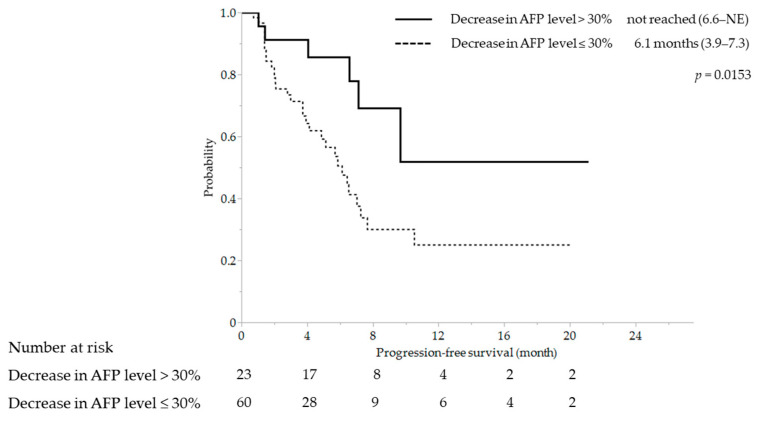
Kaplan–Meier curve for progression-free survival stratified by a decrease in AFP level in the high-AFP group. AFP, alpha-fetoprotein; NE, not estimable.

ORR was significantly different between a decrease in AFP level > 30% and ≤30% at 69.6% (16/23) and 28.3% (17/60), respectively (*p* = 0.0006). Significant differences were also observed in DCR (95.7% [21/23] vs. 68.3% [41/60], *p* = 0.0035) (Table 2). Similarly, median PFS was significantly longer with a decrease in AFP level > 30% (NE [95% CI, 7.1–NE] vs. 6.1 months [95% CI, 3.9–7.7], *p* = 0.0153) (Figure 3).

### 3.4. Predictor of Early PD in the High-AFP Group

Early PD was determined as PD at the initial imaging evaluation 6 weeks after treatment initiation. The cutoff value for early PD was set at an increase in AFP level ≥ 30% from baseline to 3 weeks after treatment initiation (sensitivity, 50.0%; specificity, 81.0%; and AUC, 0.774 by ROC curve analysis). As shown in Figure 2a, many of the patients with an increase in AFP level ≥ 30% had PD. Univariate analysis revealed that the predictors of early PD were the presence of EHS (odds ratio, 3.478; 95% CI, 1.178–10.264; *p* = 0.0240) and an increase in AFP level ≥ 30% at 3 weeks after treatment initiation (odds ratio, 3.478; 95% CI, 1.178–10.264; *p* = 0.0240). Multivariate analysis revealed that the predictors of early PD were the presence of EHS (odds ratio, 3.682; 95% CI, 1.106–12.262; *p* = 0.0337) and an increase in AFP level ≥ 30% at 3 weeks after treatment initiation (odds ratio, 4.077; 95% CI, 1.180–14.092; *p* = 0.0264) (Table 4).

### 3.5. Predictor of OR Outcome in the Low-AFP Group

The change in AFP levels from baseline at 3 weeks was not used in the low-AFP group because no trend toward treatment effect was observed, as shown in Figure 2b. We used 40 mAU/mL as a cutoff value for the baseline DCP level [16]. The results of the univariate and multivariate analyses for OR are shown in Table 5. Univariate analysis revealed that the predictor of OR was baseline DCP level < 40 mAU/mL (odds ratio, 4.690; 95% CI, 1.574–13.912; *p* = 0.0055). Multivariate analysis showed that the baseline DCP level < 40 mAU/mL was an independent factor of OR (odds ratio, 3.978; 95% CI, 1.236–12.803; *p* = 0.0206). 

**Figure 4:**
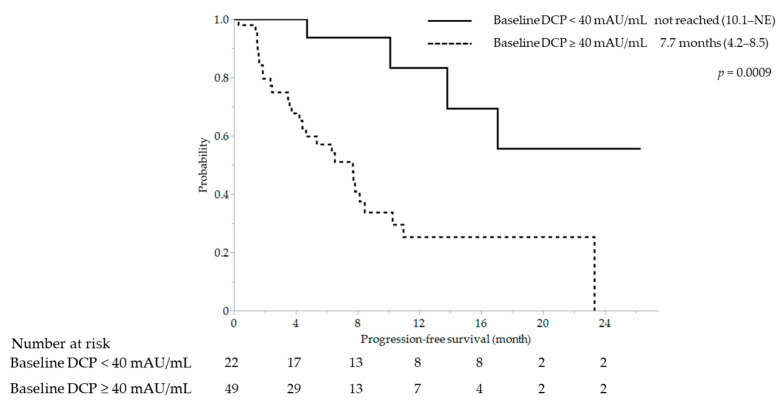
Kaplan–Meier curve for progression-free survival stratified by baseline DCP level in the low-AFP group. DCP, des-gamma-carboxy prothrombin; NE, not estimable.

ORR was significantly different between baseline DCP < 40 mAU/mL and ≥ 40 mAU/mL at 54.6% (12/22) and 20.4% (10/49), respectively (*p* = 0.0047). Significant differences were also observed in DCR (95.5% [21/22] vs. 73.5% [36/49], *p* = 0.0178) (Table 2). Median PFS was significantly better in patients with baseline DCP level < 40 mAU/mL than in those with baseline DCP level ≥ 40 mAU/mL (not reached [95% CI, 10.1–NE] vs. 7.7 months [95% CI, 4.2–8.5]; *p* = 0.0009) (Figure 4). 

### 3.6. Predictor of Early PD in the Low-AFP Group

Early PD was defined as being the same as that in the high-AFP group. Univariate analysis revealed that the predictors of early PD were late-line treatment (odds ratio, 5.417; 95% CI, 1.495–19.619; *p* = 0.0101), the presence of MVI (odds ratio, 5.357; 95% CI, 1.429–20.082; *p* = 0.0128), and up-to-seven criteria, OUT (odds ratio, 14.444; 95% CI, 1.770–117.879; *p* = 0.0127). Multivariate analysis showed that up-to-seven criteria, OUT, was an independent factor of early PD (odds ratio, 15.756; 95% CI, 1.398–177.499; *p* = 0.0257) (Table 6).

## 4. Discussion

In this study, we showed that early change in AFP levels is a predictor of OR and early PD in the high-AFP group in patients with HCC receiving atezo/bev therapy. In the low-AFP group, baseline DCP level was a predictor of OR, and the tumor burden of up-to-seven criteria was a predictor of early PD. Numerous drug therapies are available for HCC; treatment monitoring is important for appropriate sequential therapies. Tumor markers play a leading role in monitoring, with changes in AFP levels reported to be useful markers for effectively monitoring of drug therapy and local treatment [17].

The usefulness of early AFP changes in predicting treatment response has also been reported for sorafenib and lenvatinib [18,19,20]. We previously found that early reduction in AFP levels is predictive of response to lenvatinib [21]. In atezo/bev therapy, early changes in AFP level have also been reported to be useful in predicting response [22]. Zhu et al. reported that a ≥75% decrease in AFP levels at 6 weeks was a surrogate marker for imaging response [23]. Additionally, Hayakawa et al. reported that a >20% decrease in AFP levels at 6 weeks was predictive of time to progression [9]. Meanwhile, Ando et al. found that a decrease in AFP level at 3 weeks was predictive of imaging response [10]. In this study, since we found a strong correlation between the change in AFP levels at 3 and 6 weeks after atezo/bev treatment in the high-AFP group (Figure S2), the change in AFP level at 3 weeks could be used as a valuable predictor of response to treatment. Furthermore, the best cutoff value of the change in AFP levels was statistically set.

In this study, patients were categorized into two groups based on a baseline AFP of 20 ng/mL. A decrease in AFP level > 30% at 3 weeks was found to be useful for early prediction of OR in the high-AFP group. In contrast, in the low-AFP group, a baseline DCP level < 40 mAU/mL was found to be useful for early prediction of OR. Similarly, for PFS predictors, a decrease in AFP level > 30% in the high-AFP group (hazard ratio, 0.378; 95% CI, 0.148–0.970; *p* = 0.0430) and baseline DCP level < 40 mAU/mL in the low-AFP group (hazard ratio, 0.171; 95% CI, 0.056–0.520; *p* = 0.0019) were independent factors in the multivariate analysis (Tables S1 and S2). Independent factors for OS were the absence of MVI (hazard ratio, 0.422; 95% CI, 0.192–0.926; *p* = 0.0314) in the high-AFP group and first-line treatment (hazard ratio, 0.240; 95% CI, 0.079–0.729; *p* = 0.0118), mALBI 1–2a (hazard ratio, 0.138; 95% CI, 0.040–0.482; *p* = 0.0019), and the absence of EHS (hazard ratio, 0.125; 95% CI, 0.024–0.646; *p* = 0.0128) in the low-AFP group in the multivariate analysis. Although a decrease in AFP level in the high-AFP group and baseline DCP level in the low-AFP group were not significant factors, both factors demonstrated significant trends (Tables S3 and S4).

To the best of our knowledge, this is the first study to examine the prediction of atezo/bev treatment response categorized into two groups based on a baseline AFP of 20 ng/mL. Patients with baseline AFP level ≥ 20 ng/mL who had a decrease in AFP level > 30% after 3 weeks and patients with baseline AFP level < 20 ng/mL who had baseline DCP level < 40 mAU/mL had significantly higher response rates and significantly longer PFS. AFP and DCP are considered good markers because they can be easily measured using conventional tests and are valuable for early predicting prognosis.

In the IMbrave150 trial, early PD, assessed per RECIST1.1, was detected in 19.3% at the first imaging evaluation 6 weeks after treatment initiation [1]. In real-world clinical practice, early PD occurs in 13.7–25.0% of patients with HCC [7,8,17,24]. In this study, 22.1% of patients had early PD per RECIST 1.1. Since the half-life of bev is approximately 3 weeks [25,26], prediction of early PD is vital for the appropriate transition to the next treatment regimen. Zhu et al. reported that an increase in AFP level > 10% was a surrogate marker for early PD [23]. This study showed that an increase in AFP level ≥ 30% at 3 weeks after treatment initiation is a useful predictor of early PD in the high-AFP group. In contrast, in the low-AFP group, tumor burden of up-to-seven criteria, OUT, was a predictor of early PD.

**Figure 5:**
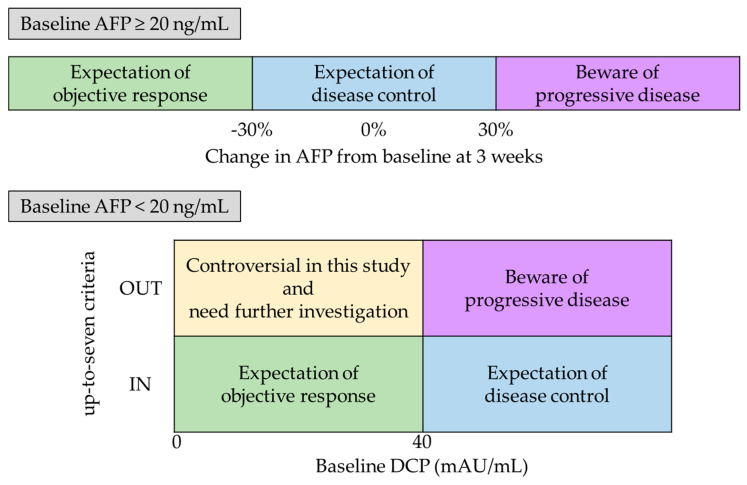
Chart of study results summary. AFP, alpha-fetoprotein; DCP, des-gamma carboxy prothrombin.

In this study, the first imaging evaluation was performed at 6 weeks, and the third atezo/bev therapy was administered on the same day. According to the study results, the possibility of early PD should be considered if patients with AFP level ≥ 20 ng/mL have an increase in AFP level ≥ 30% at 3 weeks or if patients with AFP level < 20 ng/mL have up-to-seven criteria, OUT, implying high tumor burden. Careful evaluation of images at 6 weeks is necessary for an appropriate transition to the next treatment. In contrast, a decrease in AFP level > 30% at 3 weeks in patients with baseline AFP level ≥ 20 ng/mL and baseline DCP level < 40 mAU/mL in those with baseline AFP level < 20 ng/mL may demonstrate the efficacy of the current treatment with the expectation of an imaging response. In patients with baseline AFP level < 20 ng/mL, both baseline DCP < 40 mAU/mL and up-to-seven criteria, OUT, were controversial in this study and need further investigation (Figure 5).

This study had some limitations. First, it was a retrospective study. However, it was a multicenter study with many patients, and it used tumor markers that were highly quantitative and objective factors. Second, adverse events were not evaluated in this study because of a lack of objectivity. Many immune-related adverse events (irAEs) have been reported with atezo/bev therapy [1]. Therefore, patients with a history of autoimmune disease were excluded from the clinical trials. However, the incidence of grade 3 or higher irAEs was reported to be similar in patients with and without a history of autoimmune disease in cohort studies of melanoma [27]. Conversely, severe colitis and toxicities requiring early discontinuation of therapy were reported to occur more frequently in patients with a history of inflammatory bowel disease [27,28]. Therefore, the development of irAEs should be constantly monitored during atezo/bev therapy. 

Other prognostic markers, such as pretreatment neutrophil-to-lymphocyte ratio (NLR) [29,30,31,32] and interleukin-6 (IL-6) [33], have been reported to be useful, but were not analyzed in this study. In the future, it may be necessary to consider novel markers such as NLR and IL-6, in addition to tumor markers in atezo/bev therapy.

This study provided detailed data on the therapeutic efficacy of atezo/bev for unresectable HCC. AFP trends during the first 3 weeks of treatment and baseline DCP level are useful treatment response predictors in clinical practice. We are not currently conducting a prospective study to validate the results, but would like to explore this further in the future.

## 5. Conclusions

In atezo/bev therapy for unresectable HCC, the change in AFP levels at 3 weeks in patients with baseline AFP level ≥ 20 ng/mL and baseline DCP level or tumor burden in those with AFP level < 20 ng/mL were found to predict treatment response. Thus, it appears reasonable to focus on tumor markers for early prediction of treatment response.

## Figures and Tables

**Table 1:** Patient characteristics.

		Total	AFP ≥ 20 ng/mL	AFP < 20 ng/mL
Factor		*n* = 154	*n* = 83	*n* = 71
Age (years)		75 (70–80)	74 (68–80)	76 (70–80)
Sex	Male/female	124/30	61/22	63/8
ECOG-PS	0/1/2/3	132/18/2/2	71/8/2/2	61/10/0/0
Etiology	HBV/HCV/NBNC	17/52/85	12/31/40	5/21/45
Child-Pugh class	A/B	145/9	75/8	70/1
ALBI score		−2.32 (−2.65 to −2.09)	−2.31 (−2.68 to −1.99)	−2.33 (−2.64 to −2.17)
mALBI grade	1/2a/2b/3	46/38/68/2	26/17/38/2	20/21/30/0
BCLC	A/B/C	11/79/64	5/37/41	6/42/23
MVI	Absence/presence	109/45	51/32	58/13
EHS	Absence/presence	125/29	62/21	63/8
Line	First/late	99/55	56/27	43/28
Tumor number		4 (2–10)	4 (2–10)	4 (3–7)
Tumor size	(mm)	30 (18–61)	43 (21–82)	24 (15–40)
UT7	IN/OUT	56/98	25/58	31/40
AFP	(ng/mL)	31.1 (4.8–634.0)	528.4 (136.7–6243.0)	4.4 (2.4–8.8)
DCP	(mAU/mL)	382.4 (51.7–4874.0)	868.0 (160.8–12426.0)	120.7 (29.2–1004.0)

Values are expressed as numbers, median (interquartile range); HBV, hepatitis B virus; HCV, hepatitis C virus; NBNC, non-B, non-C; ECOG-PS, Eastern Cooperative Oncology Group performance status; BCLC, Barcelona Clinic Liver Cancer; ALBI, albumin-bilirubin score; mALBI, modified ALBI; MVI, macrovascular invasion; EHS, extrahepatic spread; UT7, up-to-seven criteria; AFP, alpha-fetoprotein; DCP, des-gamma carboxy prothrombin.

**Table 2:** Relationship between the best treatment response based on RECIST 1.1 and the response of tumor markers.

Response	CR	PR	SD	PD	ORR	DCR
Total (*n* = 154)	9	46	65	34	35.7%	77.9%
High-AFP group (*n* = 83)						
Decrease in AFP level > 30%	2	14	5	2	69.6% *	95.7% **
Decrease in AFP level ≤ 30%	1	16	24	19	28.3%	68.3%
Low-AFP group (*n* = 71)						
Baseline DCP < 40 mAU/mL	2	10	9	1	54.6% ^†^	95.5% ^††^
Baseline DCP ≥ 40 mAU/mL	4	6	26	13	20.4%	73.5%

CR, complete response; PR, partial response; SD, stable disease; PD, progressive disease; ORR, objective response rate; DCR, disease control rate; AFP, alpha-fetoprotein; DCP, des-gamma-carboxy prothrombin. * *p* = 0.0006, ** *p* = 0.0035, ^†^ *p* = 0.0047, ^††^ *p* = 0.0178.

**Table 3:** Univariate and multivariate analyses for factors affecting the objective response per RECIST 1.1 in the high-AFP group.

Factors		Univariate Analysis			Multivariate Analysis		
		Odds Ratio	95% CI	*p*-Value	Odds Ratio	95% CI	*p*-Value
Age	<75/≥75 years	0.471	0.192–1.153	0.0993			
Sex	Male/female	0.726	0.271–1.947	0.5251			
ECOG-PS	0/1–3	0.614	0.180–2.097	0.4360			
Etiology	Viral/non-viral	1.806	0.740–4.410	0.1943	1.729	0.613–4.877	0.3004
Line	First/late	1.500	0.575–3.915	0.4075	1.756	0.558–5.526	0.3355
mALBI	1–2a/2b–3	0.655	0.271–1.585	0.3477	0.557	0.200–1.552	0.2634
BCLC	A–B/C	1.300	0.538–3.139	0.5597			
MVI	Absence/presence	1.807	0.714–4.575	0.2119	2.096	0.691–6.356	0.1911
EHS	Absence/presence	1.444	0.512–4.079	0.4875	1.702	0.513–5.646	0.3848
UT7	IN/OUT	1.014	0.389–2.643	0.9765	1.161	0.371–3.639	0.7975
Decrease in AFP level >30%	Yes/no	5.782	2.022–16.535	0.0011	5.517	1.773–17.165	0.0032
Baseline AFP <400 ng/mL	Yes/no	1.105	0.458–2.665	0.8243			
Baseline DCP <40 mAU/mL	Yes/no	1.630	0.376–7.062	0.5139			

ECOG-PS, Eastern Cooperative Oncology Group performance status; BCLC, Barcelona Clinic Liver Cancer; mALBI, modified albumin-bilirubin score; MVI, macrovascular invasion; EHS, extrahepatic spread; UT7, up-to-seven criteria; AFP, alpha-fetoprotein; DCP, des-gamma carboxy prothrombin; CI, confidence interval; RECIST, Response Evaluation Criteria in Solid Tumors.

**Table 4:** Univariate and multivariate analyses for factors affecting early progressive disease per RECIST 1.1 in the high-AFP group.

Factors		Univariate Analysis			Multivariate Analysis		
		Odds Ratio	95% CI	*p*-Value	Odds Ratio	95% CI	*p*-Value
Age	≥75/<75 years	0.606	0.218–1.684	0.3369			
Sex	Female/male	1.259	0.414–3.828	0.6848			
ECOG-PS	1–3/0	1.719	0.458–6.454	0.4224			
Etiology	Non-viral/viral	1.100	0.402–3.009	0.8527	1.390	0.430–4.493	0.5824
Line	Late/first	0.857	0.288–2.550	0.7817	0.656	0.171–2513	0.5385
mALBI	2b–3/1–2a	0.646	0232–1.794	0.4017	0.606	0.187–1.969	0.4048
BCLC	C/A–B	2.321	0.817–6.598	0.1141			
MVI	Presence/absence	1.864	0.673–5.159	0.2308	2.344	0.700–7.855	0.1673
EHS	Presence/absence	3.478	1.178–10.264	0.0240	3.682	1.106–12.262	0.0337
UT7	OUT/IN	1.008	0.336–3.019	0.9892	0.741	0.202–2.719	0.6514
Increase in AFP level ≥30%	Yes/no	3.478	1.178–10.264	0.0240	4.077	1.180–14.092	0.0264

ECOG-PS, Eastern Cooperative Oncology Group performance status; BCLC, Barcelona Clinic Liver Cancer; mALBI, modified albumin-bilirubin score; MVI, macrovascular invasion; EHS, extrahepatic spread; UT7, up-to-seven criteria; AFP, alpha-fetoprotein; CI, confidence interval; RECIST, Response Evaluation Criteria in Solid Tumors.

**Table 5:** Univariate and multivariate analyses for factors affecting the objective response per RECIST 1.1 in the low-AFP group.

Factors		Univariate Analysis			Multivariate Analysis		
		Odds Ratio	95% CI	*p*-Value	Odds Ratio	95% CI	*p*-Value
Age	<75/≥75 years	1.227	0.448–3.362	0.6904			
Sex	Male/female	0.720	0.156–3.321	0.6733			
ECOG-PS	0/1	1.951	0.379–10.050	0.4241			
Etiology	Viral/non-viral	1.719	0.614–4.814	0.3027	1.600	0.487–5.232	0.4394
Line	First/late	1.607	0.556–4.642	0.3806	0.827	0.248–2.759	0.7567
mALBI	1–2a/2b–3	1.426	0.506–4.015	0.5017	1.053	0.326–3.402	0.9306
BCLC	A–B/C	0.578	0.695–4.955	0.3067			
MVI	Absence/presence	2.895	0.584–14.352	0.1932	2.066	0.370–11.537	0.4085
EHS	Absence/presence	0.400	0.090–1.774	0.2280	0.352	0.063–1.952	0.2322
UT7	IN/OUT	1.895	0.685–5.239	0.2181	1.221	0.379–3.927	0.9306
Baseline DCP <40 mAU/mL	Yes/no	4.680	1.574–13.912	0.0055	3.978	1.236–12.803	0.0206

ECOG-PS, Eastern Cooperative Oncology Group performance status; BCLC, Barcelona Clinic Liver Cancer; mALBI, modified albumin-bilirubin score; MVI, macrovascular invasion; EHS, extrahepatic spread; UT7, up-to-seven criteria; DCP, des-gamma carboxy prothrombin; CI, confidence interval; RECIST, Response Evaluation Criteria in Solid Tumors.

**Table 6:** Univariate and multivariate analyses for factors affecting early progressive disease per RECIST 1.1 in the low-AFP group.

Factors		Univariate Analysis			Multivariate Analysis		
		Odds Ratio	95% CI	*p*-Value	Odds Ratio	95% CI	*p*-Value
Age	≥75/<75 years	0.586	0.180–1.908	0.3749			
Sex	Female/male	1.417	0.254–7.907	0.6913			
ECOG-PS	1/0	1.021	0.192–5.440	0.9807			
Etiology	Non-viral/viral	1.571	0.439–5.631	0.4876	1.279	0.218–7.502	0.7850
Line	Late/first	5.417	1.495–19.619	0.0101	4.720	0.040–1.115	0.0671
mALBI	2b–3/1–2a	3.086	0.912–10.436	0.0699	2.924	0.579–14.773	0.1941
BCLC	C/A–B	1.765	0.531–5.865	0.3540			
MVI	Presence/absence	5.357	1.429–20.082	0.0128	3.556	0.638–19.813	0.1477
EHS	Presence/absence	2.836	0.589–13.664	0.1937	9.806	0.572–167.988	0.1665
UT7	OUT/IN	14.444	1.770–117.879	0.0127	15.756	1.398–177.499	0.0257
Baseline DCP ≥40 mAU/mL	Yes/no	7.583	0.925–62.172	0.0591	4.436	0.364–54.044	0.2428

ECOG-PS, Eastern Cooperative Oncology Group performance status; BCLC, Barcelona Clinic Liver Cancer; mALBI, modified albumin-bilirubin score; MVI, macrovascular invasion; EHS, extrahepatic spread; UT7, up-to-seven criteria; DCP, des-gamma carboxy prothrombin; CI, confidence interval; RECIST, Response Evaluation Criteria in Solid Tumors.

## Data Availability

All data relevant to the study are included in this article and its supplementary materials. Further inquiries can be directed to the corresponding author (I.S.).

## Supplementary Materials

The following supporting information can be downloaded at: https://www.mdpi.com/article/10.3390/cancers15112927/s1, Figure S1: Kaplan–Meier curve for (a) overall survival and (b) progression-free survival of all patients; Figure S2: Correlation analyses between change in AFP at 3 weeks and those at 6 weeks. (a) baseline AFP ≥ 20 ng/mL, (b) baseline AFP < 20 ng/mL; Table S1: Univariate and multivariate analyses for factors affecting progression-free survival in the high-AFP group; Table S2: Univariate and multivariate analyses for factors affecting progression-free survival in the low-AFP group; Table S3: Univariate and multivariate analyses for factors affecting overall survival in the high-AFP group; Table S4: Univariate and multivariate analyses for factors affecting overall survival in the low-AFP group.
