# Supplementary material for: Early Prediction of Response Focused on Tumor Markers in Atezolizumab plus Bevacizumab Therapy for Hepatocellular Carcinoma
Source: Cancers (Basel). 2023 May 26;15(11):2927. doi: 10.3390/cancers15112927 (PMC10251947; doi:10.3390/cancers15112927)
Supplement: Supplementary file 1 [file cancers-15-02927-s001.zip › Figure S2.pdf]

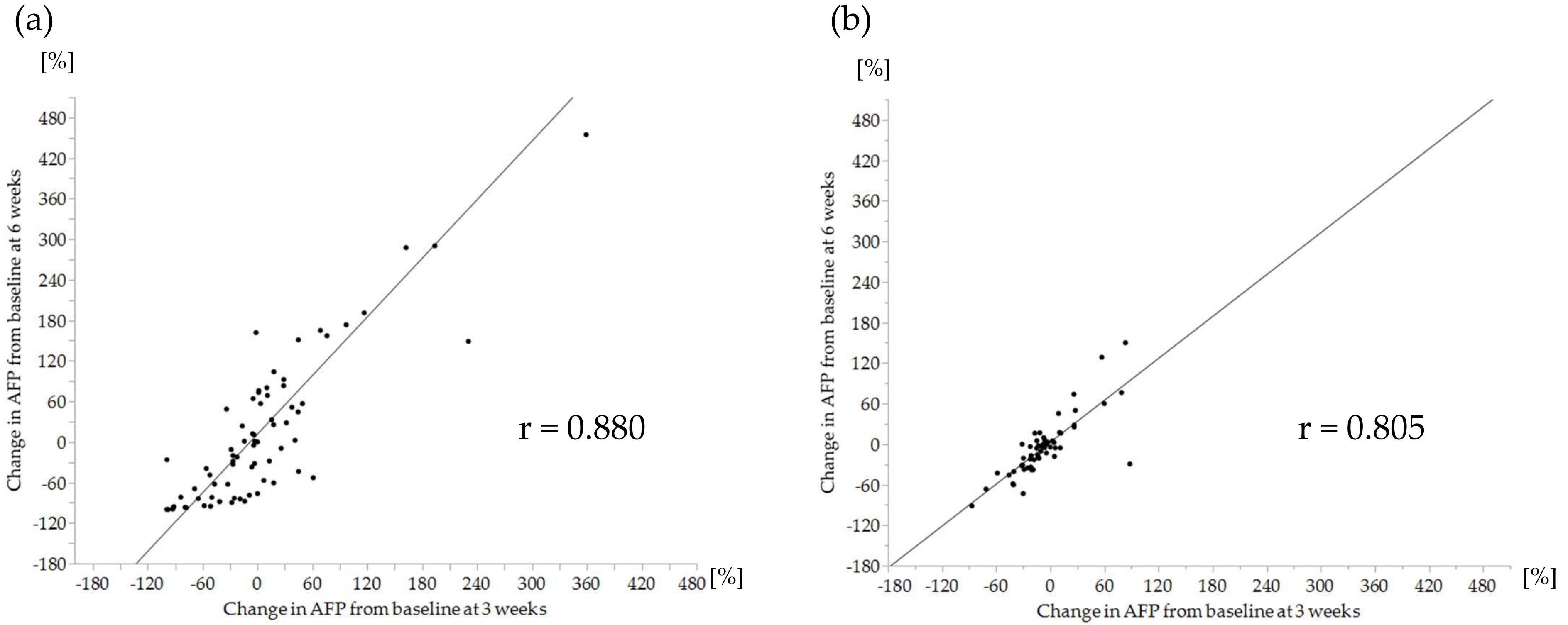

Figure S2. Correlation analysis between change in AFP at 3 weeks and those at 6 weeks. (a) baseline AFP ≥ 20 ng/mL, (b) baseline AFP < 20 ng/mL
