# Supplementary material for: Early Prediction of Response Focused on Tumor Markers in Atezolizumab plus Bevacizumab Therapy for Hepatocellular Carcinoma
Source: Cancers (Basel). 2023 May 26;15(11):2927. doi: 10.3390/cancers15112927 (PMC10251947; doi:10.3390/cancers15112927)
Supplement: Supplementary file 1 [file cancers-15-02927-s001.zip › Table S1.pdf]

Table S1. Univariate and multivariate analyses for factors affecting progression-free survival in the high-AFP group.

Factors		Univariate Analysis			Multivariate Analysis		
		Hazard ratio	95% CI	*p*-value	Hazard ratio	95%CI	*p*-value
Age	< 75/≥ 75 years	1.248	0.646–2.410	0.5098			
Sex	Male/female	0.802	0.405–1.588	0.5265			
ECOG-PS	0/1–3	0.648	0.284–1.479	0.3023			
Etiology	Viral/non-viral	0.754	0.393–1.447	0.3957	0.860	0.413–1.789	0.6863
Line	First/late	0.850	0.432–2.312	0.6387	0.806	0.380–1.709	0.5738
mALBI	1–2a/2b–3	1.511	0.780–2.926	0.2207	1.550	0.791–3.037	0.2015
BCLC	A–B/C	0.890	0.464–1.705	0.7251			
MVI	Absence/presence	0.974	0.495–1.914	0.9383	0.813	0.374–1.766	0.6013
EHS	Absence/presence	1.027	0.466–2.263	0.9436	0.827	0.366–1.869	0.6487
UT7	IN/OUT	1.371	0.704–2.671	0.3535	1.247	0.588–2.647	0.5649
Decrease in AFP level > 30%	Yes/no	0.355	0.147–0.854	0.0208	0.378	0.148–0.970	0.0430
Baseline AFP < 400 ng/mL	Yes/no	0.852	0.445–1.632	0.6297			
Baseline DCP < 40 mAU/mL	Yes/no	0.917	0.323–2.603	0.8714			

ECOG-PS, Eastern Cooperative Oncology Group performance status; BCLC, Barcelona Clinic Liver Cancer; mALBI, modified albumin-bilirubin score; MVI, macrovascular invasion; EHS, Extrahepatic spread; UT7, up-to-seven criteria; AFP, alpha-fetoprotein; DCP, des-gamma carboxy prothrombin; CI, confidence interval
