# Supplementary material for: Early Prediction of Response Focused on Tumor Markers in Atezolizumab plus Bevacizumab Therapy for Hepatocellular Carcinoma
Source: Cancers (Basel). 2023 May 26;15(11):2927. doi: 10.3390/cancers15112927 (PMC10251947; doi:10.3390/cancers15112927)
Supplement: Supplementary file 1 [file cancers-15-02927-s001.zip › Table S2.pdf]

Table S2. Univariate and multivariate analyses for factors affecting progression-free survival in the low-AFP group.

Factors		Univariate Analysis			Multivariate Analysis		
		Hazard ratio	95% CI	*p*-value	Hazard ratio	95%CI	*p*-value
Age	< 75/≥ 75 years	0.939	0.461–1.912	0.8621			
Sex	Male/female	0.822	0.285–2.368	0.7161			
ECOG-PS	0/1	2.054	0.483–8.735	0.3299			
Etiology	Viral/non-viral	0.508	0.233–1.105	0.0878	0.513	0.218–1.208	0.1268
Line	First/late	0.354	0.173–0.725	0.0046	0.430	0.202–0.917	0.0288
mALBI	1–2a/2b	0.539	0.268–1.080	0.0814	0.872	0.411–1.851	0.7217
BCLC	A–B/C	1.358	0.607–3.038	0.4570			
MVI	Absence/presence	0.392	0.179–0.858	0.0191	0.484	0.217–1.077	0.0752
EHS	Absence/presence	0.870	0.264–2.870	0.8189	1.303	0.318–5.341	0.7133
UT7	IN/OUT	0.280	0.119–0.656	0.0034	0.280	0.108–0.728	0.0091
Baseline DCP < 40 mAU/mL	Yes/no	0.199	0.069–0.572	0.0027	0.171	0.056–0.520	0.0019

ECOG-PS, Eastern Cooperative Oncology Group performance status; BCLC, Barcelona Clinic Liver Cancer; mALBI, modified albumin-bilirubin score; MVI, macrovascular invasion; EHS, Extrahepatic spread; UT7, up-to-seven criteria; DCP, des-gamma carboxy prothrombin; CI, confidence interval
