# Supplementary material for: Early Prediction of Response Focused on Tumor Markers in Atezolizumab plus Bevacizumab Therapy for Hepatocellular Carcinoma
Source: Cancers (Basel). 2023 May 26;15(11):2927. doi: 10.3390/cancers15112927 (PMC10251947; doi:10.3390/cancers15112927)
Supplement: Supplementary file 1 [file cancers-15-02927-s001.zip › Table S3.pdf]

Table S3. Univariate and multivariate analyses for factors affecting overall survival in the high-AFP group.

Factors		Univariate Analysis			Multivariate Analysis		
		Hazard ratio	95% CI	*p*-value	Hazard ratio	95%CI	*p*-value
Age	< 75/≥ 75 years	0.709	0.352–1.426	0.3346			
Sex	Male/female	1.138	0.537–2.413	0.7351			
ECOG-PS	0/1–3	0.543	0.221–1.332	0.1820			
Etiology	Viral/non-viral	1.363	0.662–2.808	0.4003	2.066	0.913–4.675	0.0816
Line	First/late	1.469	0.709–3.044	0.3014	1.372	0.626–3.008	0.4292
mALBI	1–2a/2b–3	0.833	0.415–1.672	0.6070	0.937	0.440–1.996	0.8656
BCLC	A–B/C	0.431	0.209–0.889	0.0226			
MVI	Absence/presence	0.463	0.231–0.930	0.0305	0.422	0.192–0.926	0.0314
EHS	Absence/presence	0.434	0.189–0.998	0.0495	0.588	0.242–1.431	0.2419
UT7	IN/OUT	1.070	0.505–2.267	0.8605	1.081	0.466–2.507	0.8552
Decrease in AFP level > 30%	Yes/no	0.533	0.205–1.386	0.1967	0.430	0.159–1.162	0.0961
Baseline AFP < 400 ng/mL	Yes/no	0.759	0.373–1.543	0.4457			
Baseline DCP < 40 mAU/mL	Yes/no	0.802	0.242–2.666	0.7194			

ECOG-PS, Eastern Cooperative Oncology Group performance status; BCLC, Barcelona Clinic Liver Cancer; mALBI, modified albumin-bilirubin score; MVI, macrovascular invasion; EHS, Extrahepatic spread; UT7, up-to-seven criteria; AFP, alpha-fetoprotein; DCP, des-gamma carboxy prothrombin; CI, confidence interval
