# Supplementary material for: Early Prediction of Response Focused on Tumor Markers in Atezolizumab plus Bevacizumab Therapy for Hepatocellular Carcinoma
Source: Cancers (Basel). 2023 May 26;15(11):2927. doi: 10.3390/cancers15112927 (PMC10251947; doi:10.3390/cancers15112927)
Supplement: Supplementary file 1 [file cancers-15-02927-s001.zip › Table S4.pdf]

Table S4. Univariate and multivariate analyses for factors affecting overall survival in the low-AFP group.

Factors		Univariate Analysis			Multivariate Analysis		
		Hazard ratio	95% CI	*p*-value	Hazard ratio	95%CI	*p*-value
Age	< 75/≥ 75 years	1.495	0.605–3.694	0.3833			
Sex	Male/female	0.778	0.229–2.721	0.7076			
ECOG-PS	0/1	0.841	0.245–2.891	0.7838			
Etiology	Viral/non-viral	0.618	0.241–1.588	0.3180	0.660	0.233–1.865	0.4325
Line	First/late	0.282	0.107–0.745	0.0106	0.240	0.079–0.729	0.0118
mALBI	1–2a/2b	0.270	0.102–0.713	0.0082	0.138	0.040–0.482	0.0019
BCLC	A–B/C	0.602	0.235–1.544	0.2909			
MVI	Absence/presence	0.502	0.180–1.398	0.1869	1.305	0.413–4.119	0.6503
EHS	Absence/presence	0.296	0.080–1.095	0.0682	0.125	0.024–0.642	0.0128
UT7	IN/OUT	0.332	0.110–1.002	0.0682	0.469	0.143–1.532	0.2099
Baseline DCP < 40 mAU/mL	Yes/no	0.110	0.015–0.826	0.0319	0.152	0.019–1.197	0.0736

ECOG-PS, Eastern Cooperative Oncology Group performance status; BCLC, Barcelona Clinic Liver Cancer; mALBI, modified albumin-bilirubin score; MVI, macrovascular invasion; EHS, Extrahepatic spread; UT7, up-to-seven criteria; DCP, des-gamma carboxy prothrombin; CI, confidence interval
